# Fish Meal Replacement by Mixed Plant Protein in the Diets for Juvenile Yellow Catfish *Pelteobagrus fulvidraco*: Effects on Growth Performance and Health Status

**DOI:** 10.1155/2022/2677885

**Published:** 2022-11-22

**Authors:** Ya-Kang Han, Yi-Chuang Xu, Zhi Luo, Tao Zhao, Hua Zheng, Xiao-Ying Tan

**Affiliations:** Laboratory of Nutrition and Feed Formulation for Aquatic Economic Animals, Huazhong Agricultural University, Wuhan 430070, China

## Abstract

Increasing dietary replacement levels of fish meal by alternative plant proteins are of value for aquaculture. Here, a 10-week feeding experiment was undertaken to explore the effects of fish meal replacement by mixed plant protein (at a 2 : 3 ratio of cottonseed meal to rapeseed meal) on growth performance, oxidative and inflammatory responses, and mTOR pathway of yellow catfish *Pelteobagrus fulvidraco*. Yellow catfish (2.38 ± 0.1 g, mean ± SEM) were randomly divided into 15 indoors fiberglass tanks, 30 fish each tank, and fed five isonitrogenous (44% crude protein) and isolipidic (9% crude fat) diets with fish meal replaced by mixed plant protein at 0% (the control), 10% (RM10), 20% (RM20), 30% (RM30), and 40% (RM40), respectively. Among five groups, fish fed the control, and RM10 diets tended to have higher growth performance, higher protein content, and lower lipid content in livers. Dietary mixed plant protein substitute increased hepatic free gossypol content and damaged liver histology and reduced the serum total essential amino acids, total nonessential amino acids, and total amino acid contents. Yellow catfish fed the control, and RM10 diets tended to have higher antioxidant capacity. Dietary mixed plant protein replacement tended to promote proinflammatory responses and inhibited mTOR pathway. Based on the second regression analysis of SGR against mixed plant protein substitutes, the optimal replacement level of fish meal by mixed plant protein was 8.7%.

## 1. Introduction

Fish meal (FM) is thought to be a high-quality protein source in aquatic feeds because it possesses good palatability, well-balanced essential amino acid profiles, and essential fatty acids which are required by fish [1]. Therefore, fish meal is most popular protein source in aquatic feeds. However, because of its increasing demand, reduced supplies, and increasing price, scientists and feed manufacturers are searching for alternative dietary protein sources to replace fish meal [2, 3]. Based on the annual yield, cottonseed meal and rapeseed meal are thought to rank the second and third plant protein sources, respectively, and they had relatively high protein content, abundant resources, low price, and convenient processing.

The application of cottonseed meal and rapeseed meal to replace fish meal has been assessed for many fish species [4–7]. But single safe levels of cottonseed meal and rapeseed meal added in aquaculture diets were limited because of the amino acid imbalance and antinutritional factors (ANFs), such as protease inhibitors, phytic acid, tannins, and glucosinolates (GLS), which adversely influence their utilization in aquaculture feed [5, 8, 9]. More and more researches reported that the replacement of multiple plant protein sources for fish meal can partially alleviate amino acid imbalance of single protein source, and accordingly increased the replacement level [10, 11]. Therefore, in present study, we hypothesize that the mixed plant proteins can increase their replacement level for fish meal.

Several antioxidant enzymes, such as superoxide dismutase (SOD) and catalase (CAT), play important roles in oxidative stress. SOD is responsible for the conversion of superoxide radicals to hydrogen peroxide and CAT for hydrogen peroxide to water and oxygen [12]. Furthermore, the antioxidant enzyme activities (including SOD and CA T) are related to their gene transcription, which is controlled by the Kelch-like-ECH-associated protein 1 (Keap1) and the NF-E2-related nuclear factor 2 (Nrf2) [13, 14]. On the other hand, the inflammatory responses were mediated by cytokines and crucial components for the cellular immune response [15]. Tumor necrosis factor-*α* (TNF-*α*) and interleukin 8 (*IL8*) are the important proinflammatory cytokines [16, 17], and transforming growth factor-*β* (TGF-*β*) and interleukin 10 (*il10*) are the important anti-inflammatory cytokines [18]. On the other hand, growth performance is mainly derived from cell proliferation and growth, which are regulated by mammalian target of rapamycin (mTOR) pathway [19]. The mTOR pathway is considered an integration point between the growth and protein metabolism [20]. The pathway regulates protein synthesis via the eukaryotic translation initiation factor 4e-binding protein (4E-BP) and ribosomal protein S6 kinase (S6K1) [21, 22]. Dietary fish meal replacement with a high proportion of cottonseed meal or rapeseed meal could lead to liver inflammation, reduce the antioxidant capacity, and cause tissue damage in fish [23–25]. Studies suggested that high inclusion levels of cottonseed meal and rapeseed meal reduced growth performance and feed utilization [8, 9]. Studies also suggested that plant proteins can significantly inhibit the mTOR pathway [26, 27]. These negative effects greatly limit the level of plant protein in aquafeeds. Therefore, how to improve their inclusion levels in aquaculture feed seems very urgent [10]. Yellow catfish *Pelteobagrus fulvidraco* is a very important freshwater culture fish in China and other Asian countries [28]. Because of its good fillet quality, yellow catfish has promising market prospects in Asia. The increasing price of fish meal and the shortage of fish meal resources became the main factor limiting its wide use in aquatic feeds. Therefore, it is important to search for the alternative plant protein sources to replace dietary fish meal to reduce feed cost, improve economic benefit, and promote the development of yellow catfish industry. The experiment was to evaluate the effects of fish meal replacement with the mixed plant protein on growth performance, antioxidant and inflammation response, and mTOR pathway of yellow catfish.

## 2. Materials and Methods

### 2.1. Ethic Statement, Diet Preparation, and Fish Culture

The present study on yellow catfish conforms to the ethical guideline of Huazhong Agriculture University and is approved by the Ethics committee of the University.

Five isonitrogenous (44% protein) and isolipidic (9% lipid) diets were formulated by replacing 0% (the control), 10% (RM10), 20% (RM20), 30% (RM30), and 40% (RM40) of fish meal protein with mixed plant proteins, respectively (Table 1). The mixed plant proteins were produced at a 3 : 2 ratio of rapeseed meal to cottonseed meal, based on Jiang et al. [29]. The dry ingredients were ground and sieved through the 40-mesh screen. Then, dry feedstuffs were weighed accurately according to the formula and thoroughly mixed. After addition of oil and water, the compound was pelleted using a meat grinder with 10 mm sieve. The feeds were dried in a forced air circulation oven at 60°C. The dry pellets were placed in plastic bags and kept at −20°C until feeding. The formula and proximate composition of diets are shown in Table 1.

Then, they were mixed thoroughly and cut into pellets with the pellet presser. All five experimental diets were dried at 60°C in the oven and stored at −20°C.

The present study followed the institutional ethics guidelines of the Ethics Committee of Huazhong Agricultural University (HZAU). The experimental protocols were approved by the Ethical Committee of HZAU. Juvenile yellow catfish were purchased from the local fish farm and randomly stocked in 15 tanks (300-L in water volume) for 2 weeks of acclimation. During the acclimatization, yellow catfish were fed to apparent satiation twice a day (08:30 and 16:30) with RM20 diets. When the feeding experiment has begun, yellow catfish were fasted for 24 h before weighing. Then, similar size and healthy yellow catfish (2.38 ± 0.10 g) were stocked to 15 tanks (300 L in water volume), 30 fish per tank. Each diet was assigned to the three tanks. The experimental tanks were provided with the dechlorinated tap water and with continuous aeration to maintain the dissolved oxygen (DO) level above the saturation. The fish were hand fed to the satiation twice daily (0830 and 1630 h). Yellow catfish were weighed every 2 weeks. The feed intake in each tank was recorded daily. The feces were removed before the feeding in the morning. The experimental tanks were cleaned every 2 weeks when yellow catfish were removed for weighing. Mortality was recorded daily. The feeding experiment lasted for 10 weeks. During the feeding trial, water quality was monitored twice every week, and showed below: water temperature from 28.3 to 29.4°C; DO 6.37-6.67 mg/L and NH_4_-N not higher than 0.1 mg/L.

### 2.2. Growth Performance, Biological Indices, and Sampling

At the end of the feeding trial, yellow catfish were fasted for 24 h before sampling. They were euthanized with MS-222 (100 mg/L water) and weighed to determine weight gain (WG) and specific growth rate (SGR). Then, 6 fish were randomly selected from each tank, and the blood and liver were immediately collected. The livers were frozen in the liquid N_2_ and stored at −80°C for the RNA and protein isolation. The blood was centrifuged at 3500 g min^−1^ for 10 min, and the serum was used to determine the free amino acids content. Another three fish per tank were selected, and the livers were collected, and then fixed in 4% paraformaldehyde for histological observation. For analyzing enzyme activity, another six fish per tank were selected, and the liver was sampled, frozen in liquid nitrogen and stored at −80°C for the subsequent analysis.

### 2.3. Proximate Composition and Free Gossypol Content

The diets and liver proximate composition were analyzed by AOAC [30] standard method. Briefly, the dry matter was analyzed by drying the samples at 105°C until the constant weight was achieved. Crude protein and lipid contents were determined using the Kjeldahl method and the Soxhlet ether method, respectively. Ash content was analyzed after the samples were burned for 8 h in the muffle furnace at 550°C.

The contents of free gossypol in diets and liver were determined by the aniline method. Briefly, gossypol was extracted in the presence of a mixture of 2-propanol, 3-amino-1-propanol and hexane. Then, gossypol was converted into the gossypol-aniline. Finally, the absorbance of the compound was measured at the wavelength of 440 nm.

### 2.4. Analysis for Free Amino Acids Contents

Serum samples were deproteinized by mixing thoroughly with 10% sulfosalicylic acid solution, followed by the incubation at 4°C for 1 h. They were then centrifuged at 13000 rpm for 15 min. Finally, the supernatant was passed through a 0.22-*μ*m filter and then used for amino acid analysis via the automated amino acid analyzer (A300-advanced Autoanalyzer, MembraPure, Germany).

### 2.5. Antioxidant Enzymatic Activities and Lipid Peroxidation Analysis

The activities of antioxidant enzymes, such as total T-SOD, CAT and total antioxidant capacity (T-AOC), and lipid peroxidation (MDA) were measured according to our recent study [31]. The liver tissues were homogenized in ice-cold phosphate buffered saline (PBS). The activities of antioxidant enzymes (T-SOD and CAT), total antioxidant capacity (T-AOC), and MDA were determined by commercial reagent kits (Jiancheng, Nanjing, China). One unit of enzyme activity was defined as the amount of enzyme which converts 1 *μ*M substrate to the product per minute at 37°C and expressed as units per milligram of soluble protein. MDA content was determined based on the reaction of the thiobarbituric acid, and the absorbance was detected at 535 nm with the spectrophotometer.

### 2.6. Liver Histology

Hematoxylin-Eosin (H&E) staining was conducted after Wu et al. [32]. The sections were imaged by light microscope (Olympus BX53, Tokyo, Japan), and morphological measurements were performed by the ImageJ software (version 1.51, NIH, Maryland, USA).

### 2.7. Real-Time Quantitative PCR Analysis of Gene Expression

Total RNA in the liver tissues was extracted using the TRIzol method. The integrity of RNA was evaluated by the agarose gel electrophoresis. The cDNA synthesis was performed with the UnionScript First-strand cDNA Synthesis Mix (Genesand, SR511). The gene-specific primers are given in Table 2. At first, we measured the transcriptional stabilities of 10 housekeeping genes (*β*-actin, 18S ribosomal RNA, hypoxanthine-guanine phosphoribosyltransferase, ubiquitin C, *β*2-microglobulin, tubulin-A, glyceraldehyde 3-phosphate dehydrogenase, TATA-binding protein, ribosomal protein L7, and E74-like factor-A) and determined the best combination of two genes by geNorm online tool (https://genorm.cmgg.be/). Finally, we used the 2^−ΔΔCt^ method to calculate the relative expression of genes after we normalized to their geometric mean of two genes.

### 2.8. Western Blotting

Based on the method described in our laboratory [31], we used the western blot to detect mTOR, p-mTOR, S6, p-S6, Nrf2, and Keap1 protein expression levels. Briefly, the liver was prepared with the RIPA buffer (Thermo Fisher Scientific). Then, the proteins (40 *μ*g from each sample) were separated on 8% or 12% SDS–polyacrylamide gel, depending on the molecular weight of proteins. They were transferred to the PVDF membranes (Thermo Fisher Scientific), blocked with 8% (w/v) skimmed milk in the TBST buffer (20 mM Tris–HCl, 0.1% Tween 20, 150 mM sodium chloride, pH 7.5) for 1 h, washed thrice with the TBST buffer for 10 min each, and followed by the incubation with specific primary antibodies, such as rabbit anti-p-mTOR-S2448 (1 : 2000, AP0094; Abclonal, Wuhan, China), anti-mTOR (1 : 1000, A2445; Abclonal), anti-p-S6- S235/236 (1 : 1000, AP0538; Abclonal), anti-S6 (1 : 1000, A6058; Abclonal), anti-Nrf2 (1 : 1000, A0674; Abclonal), and anti-Keap1 (1 : 5000, A17061; Abclonal) for overnight at 4°C. They were then incubated with goat anti-rabbit secondary antibody (1 : 10000). Immunoreactive bands were visualized via the enhanced chemiluminescence (Cell Signaling Technology) and quantified via the densitometry using ImageJ software (version 1.42, NIH).

### 2.9. Statistical Analysis

The experimental data are presented as mean ± standard errors of means (SEMs). We performed statistical analysis by the Prism 8 software (GraphPad Software, CA, USA). Before the statistical analysis, we verified the normality of the data using the Shapiro-Wilk test and analyzed the homogeneity of variances by Levene's test. Statistical significance was determined by the one-way ANOVA with the Duncan's post hoc test. *P* value was set at <0.05 for statistically significant differences.

## 3. Results

### 3.1. Survival, Growth Performance, and Feed Utilization

**Figure 1:**
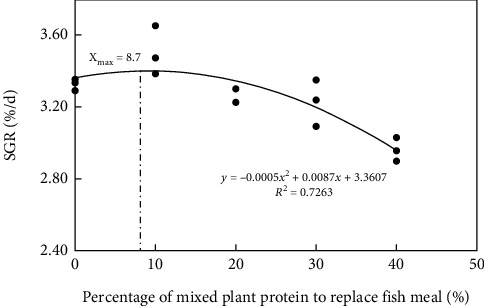
The relationship between SGR and dietary mixed plant protein replacement level in yellow catfish for 10 weeks.

In the present study, survival, growth performance and feed utilization are presented in Table 3. Among five groups, no significant difference was observed in survival rate, and WG (weight gain) and SGR (specific growth rate) were the highest for the RM10 group, and FCR (feed conversion ratio) and FI (feed intake) were highest for the RM40 group. Based on the second-regression analysis model between SGR and dietary mixed plant protein replacement levels, their optimal substitution level for fish meal was 8.7% (Figure 1).

### 3.2. Proximate Composition and Free Gossypol Contents

The approximate composition and free gossypol contents in the liver tissue were shown in Table 4. The crude protein content was highest for yellow catfish fed the RM10 diet, followed by the control, and showed no marked discrepancies among other three groups. The crude lipid content was highest for yellow catfish fed the RM40 diet and lowest for the control. The moisture content presented no marked differences among five dietary groups. Hepatic free gossypol content increased with dietary mixed plant protein levels.

### 3.3. Free Amino Acid Profiles in the Serum

Dietary mixed plant protein source replacement significantly influenced the free amino acid profiles in the serum (Table 5). For these essential amino acids, among the five dietary groups, yellow catfish fed the RM10 diet tended to possess higher contents of Met and Leu, and yellow catfish fed the control had higher contents of Lys, Arg, and His; in contrast, yellow catfish fed the RM40 diet had the lowest contents of Met, Lys, Val, His, Leu, Ile, Phe, and Thr. For these nonessential amino acids, among five groups, yellow catfish fed the control had higher contents of Ala, Asn, and Asp, and fish fed the RM10 diet had higher Glu content; in contrast, yellow catfish fed the RM40 diet had lowest contents of Ser, Ala, Gly, Tyr, Asn, Glu, Pro, and Asp. Total NEAA, total EAA, and total amino acid contents declined with increasing mixed plant protein replacement levels.

### 3.4. Histology

**Figure 2:**
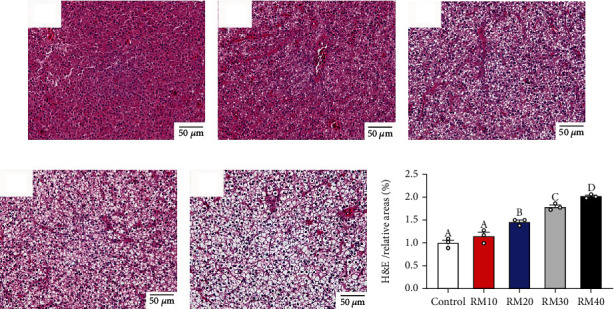
Liver tissue cross-sectional view of the five groups (H&E staining, ×400). (a) The control. (b) RM10 group. (c) RM20 group. (d) RM30 group. (e) RM40 group. (f) Relative areas for hepatic vacuoles in H&E staining.

Liver histological observation indicated that dietary mixed plant protein replacement tended to increase the vacuolation amounts (Figures 2(a) and 2(b)). Generally, no significant histological changes in the liver were found between the control and RM10 group since these fish between the two groups had the minimal vacuolation and compact hepatocytes with the nuclear in their centers (Figures 2(a) and 2(b)). However, fish fed the RM40 diet resulted in significant pathological changes of liver tissue, such as severe destruction and disarrangement of hepatocytes, and more amounts of vacuolation in hepatocytes with nuclear located beside the cell membrane (Figure 2).

### 3.5. Indicators of Antioxidant Indices in the Liver

**Figure 3:**
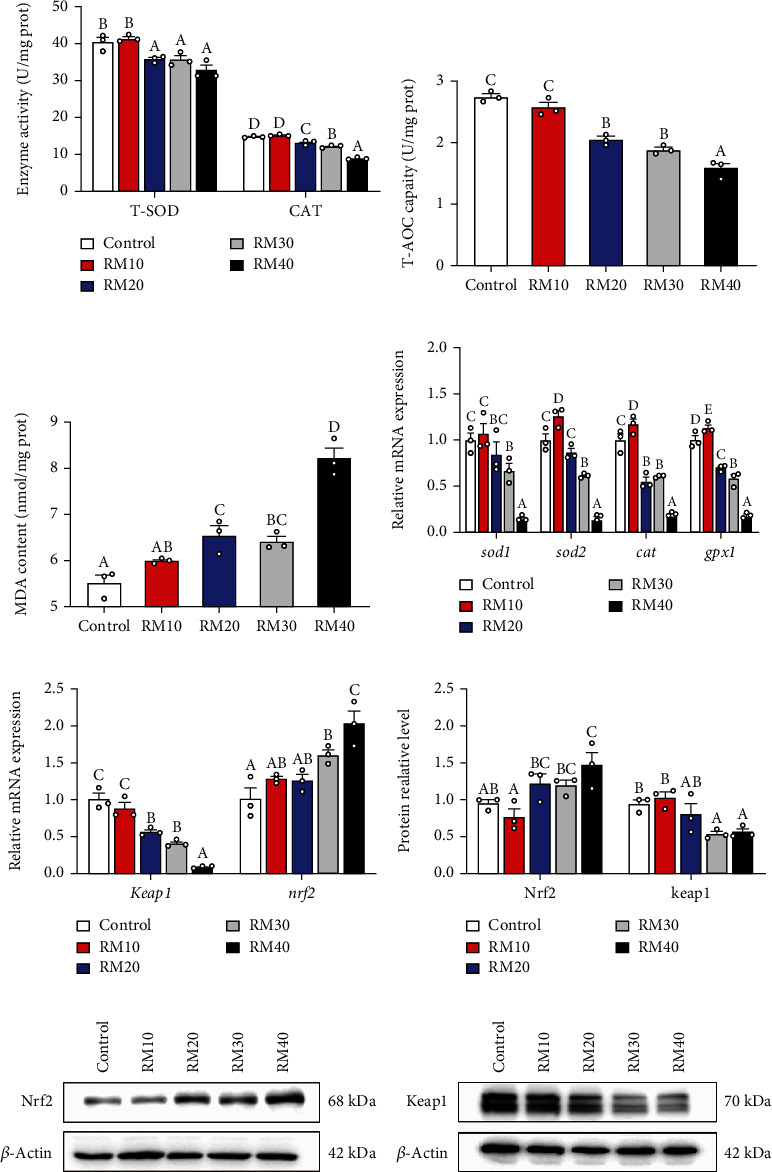
Effects of fish meal replacement by mixed plant protein sources on oxidative stress and antioxidant response in the liver of yellow catfish. (a) Activity of oxidative enzymes. (b) Total antioxidant capacity. (c) MDA content. (d) Relative mRNA expression of oxidative stress-related genes. (e) Relative mRNA expression of Nrf2 and Keap1. (f) Protein relative level of Nrf2 and Keap1. (g) Western blot of Nrf2. (h) Western blot of Keap1.Values are means ± S.E.M. (*n* = 3). Letters (a–d) denote significance *P* < 0.05 (One-way ANOVA, Duncan post hoc test).

Dietary mixed plant protein replacement significantly influenced antioxidant responses in the livers of yellow catfish (Figure 3). Among five groups, yellow catfish fed the control, and RM10 diet had the highest activities of T-SOD and CAT and total antioxidant capacity (Figures 3(a) and 3(b)). In contrast, MDA contents were the lowest for yellow catfish fed the control and RM10 diets and highest for yellow catfish fed RM40 diet (Figure 3(c)). The *sod1* mRNA levels were highest for yellow catfish fed the control and RM10 diet and lowest for yellow catfish fed the RM40 diets. The mRNA levels of *sod2*, *cat*, and *gpx1* were the highest in fish fed the RM10 diet and lower in yellow catfish fed the RM40 diet (Figure 3(d)). The *keap1* mRNA expression declined but *nrf2* mRNA expression tended to increase with increasing dietary mixed protein replacement levels (Figure 3(e)). Compared to other three groups, yellow catfish fed the control and RM10 diet had lowest Nrf2 protein expression but highest Keap1 protein expression (Figures 3(f)–3(h)).

### 3.6. The mRNA Levels of Inflammation-Related Genes of Yellow Catfish

**Figure 4:**
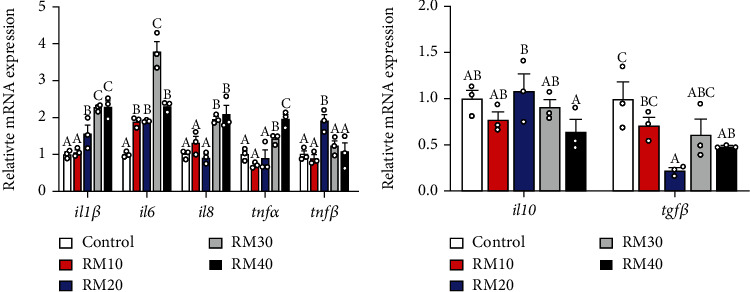
Effects of fish meal replacement by mixed plant protein on inflammatory response in the liver of yellow catfish. (a) Relative mRNA expression of proinflammatory factor related genes. (b) Relative mRNA expression of anti-inflammatory factor related genes. Values are means ± S.E.M. (*n* = 3). Letters (a–c) denote significance *P* < 0.05 (One-way ANOVA, Duncan post hoc test).

Dietary mixed plant protein replacement significantly influenced mRNA expression of genes relevant with inflammatory responses (Figure 4). For several proinflammatory factors, the mRNA levels of *il1β* were the lowest for yellow catfish fed the control and RM10 diet and the highest for yellow catfish fed RM30 and RM40 diets. The *il6* mRNA levels were the lowest for yellow catfish fed the control and highest for yellow catfish fed RM30 diet. The *il8* and *tnfα* mRNA levels were lower for yellow catfish fed the control, RM10, and RM20 diets than those for yellow catfish fed the RM30 and RM40 diet. The *tnfβ* mRNA levels were higher in the RM20 diet group than those in other four groups (Figure 4(a)).

For several anti-inflammatory factors, the mRNA levels of *il10* were higher in the RM20 group and presented no marked discrepancies among other four groups, and the *tgfβ* mRNA level was highest in the control and lowest in the RM20 group (Figure 4(b)).

### 3.7. The Expression of mTOR Pathway-Related Proteins in the Liver

**Figure 5:**
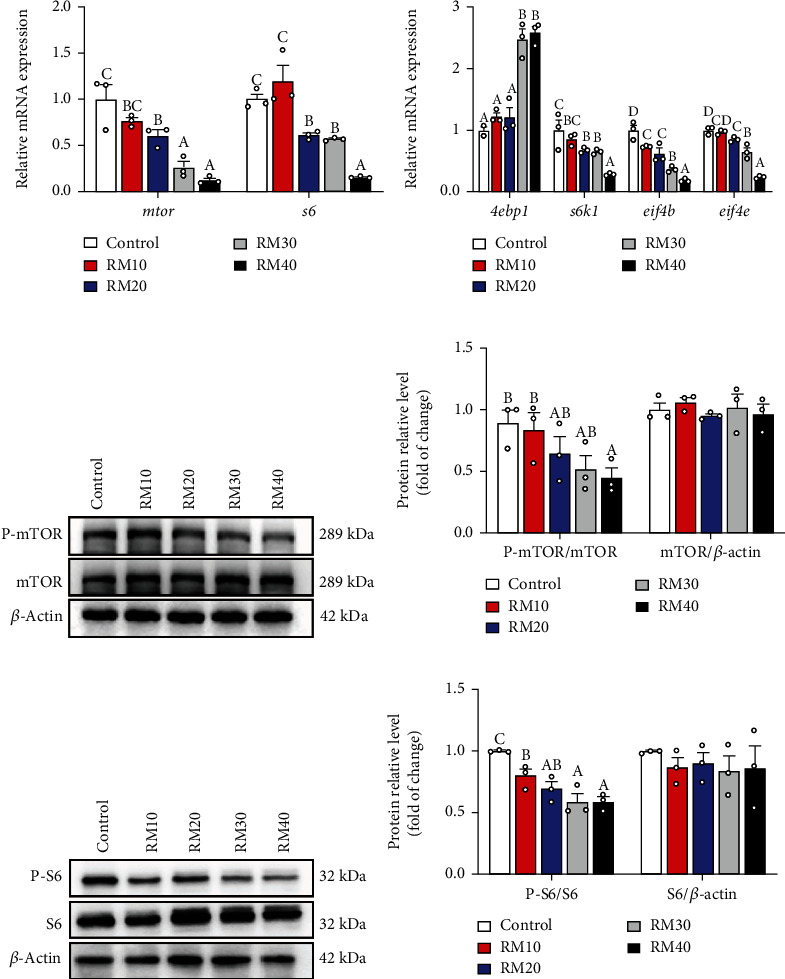
Effects of fish meal replacement by mixed plant protein on mTOR pathway in the liver of yellow catfish. (a) Western blot and protein level of mTOR and p-mTOR. (b) Western blot and protein level of S6 and p-S6. (c) Relative mRNA expression of mTOR and S6. (d) Relative mRNA expression of mTOR pathway related genes. Values are means ± S.E.M. (*n* = 3). Letters (a–d) denote significance *P* < 0.05 (One-way ANOVA, Duncan post hoc test).

Dietary mixed plant protein replacement significantly influenced mRNA and protein expression of key factors relevant with mTOR signaling pathway (Figure 5). The mTOR mRNA levels declined with the increment of plant protein replacement levels, and the s6 mRNA expression was highest for yellow catfish fed the control and RM10 diets and lowest for yellow catfish fed the RM40 diet (Figure 5(a)). The *4ebp1* mRNA levels increased, and the mRNA levels of *s6k1*, *eif4b* and *eif4e* declined with the increment of replacement levels (Figure 5(b)). Dietary protein replacement did not markedly affect the protein expression of mTOR and S6. However, the ratios of p-mTOR/mTOR and p-S6/S6 reduced with increasing dietary replacement levels (Figures 5(c)–5(f)).

## 4. Discussion

In the results of the present study, survival showed no significant differences among the treatments. Similarly, other studies suggest that high inclusion levels of cottonseed meal with high gossypol levels do not adversely influence the survival in various species [33–35]. The present study indicated that yellow catfish fed the RM10 group possessed the best growth performance and feed utilization. Moreover, compared to the control, the mixture of plant protein replaced 30% fish meal without affecting growth performance and feed utilization of yellow catfish. Many studies explored the optimal fish meal replacement level by an individual cottonseed meal or rapeseed meal in fish [9, 36]. For example, Sun et al. and Zhang et al. [9, 36] found that the growth and feed utilization of *Hemibagrus wyckioides* were reduced with increasing dietary rapeseed meal level. Previous studies found that the optimal dietary fermented cottonseed meal level to replace fish meal was 3.11% for black seabream [36]. However, Lim and Lee [6] demonstrated that the 30% dietary fish meal could be replaced by cottonseed meal for parrot fish. The discrepancies may be due to the species, feeding habitat and the composition in the diets. Different from the previous single plant protein replacement of fish meal, this study used a mixed plant protein (cottonseed meal: rapeseed meal = 2 : 3) to replace fish meal in yellow catfish diet. Compared with a single plant protein, mixed plant protein could replace more fish meal without inhibiting growth performance. Studies suggested that mixed plant proteins can alleviate the adverse influences of amino acid imbalance and antinutritional factors in a single plant protein source [10, 11]. Therefore, the mixed plant protein in fish feed was better than that of single plant protein. Studies suggested that the nutritional quality of cottonseed meal and rapeseed meal largely depends on the contents of ANFs, and the ANFs negatively influenced the activities of digestive enzymes and nutrient digestibility and growth performance of fish [9, 35]. Our study also indicated that yellow catfish fed the RM40 diet had the highest FI. Similarly, Zhou et al. [37] found that FI increased as the dietary canola meal protein increased, which could be explained by increasing the feed intake to obtain more nutrients with the increasing canola meal levels [38].

In the present study, yellow catfish fed the RM10 diet had highest crude protein content, followed by the control, and the crude protein content showed no marked discrepancies among other three groups. Similarly, studies suggested that dietary plant protein sources addition reduced body protein contents in many fish species [36, 39]. We also found that the crude lipid content was highest for yellow catfish fed the RM40 diet and lowest for yellow catfish fed the control. Alam et al. [40] found a higher lipid content in the whole body in flounder fed 75% and 100% cottonseed meal diets compared with fish fed a fish meal-based diet. Potential toxicological effects of dietary ANFs in the plant proteins may impair protein and lipid deposition of fish. Our study indicated that free gossypol content in the liver ranged between 0 and 34.42 mg/kg and increased with dietary mixed plant protein levels. The liver is the major tissue for free gossypol accumulation in fish [41]. Generally speaking, the gossypol accumulation in our study was similar to or slightly higher than those in other studies [6, 36]. However, higher hepatic gossypol contents were also reported in other studies [42, 43]. These differences support the notion that hepatic gossypol accumulation in fish can markedly be influenced by dietary types or the fish species tested. In agreement with our study, other studies suggested a positive relationship between dietary gossypol and hepatic gossypol content [42, 43]. Hardy [44] pointed out that gossypol could induce the formation of ceroid granules. Therefore, pathological changes of liver tissue of yellow catfish with increasing plant protein were likely associated with increased ANFs content.

In order to explore the absorption of dietary amino acids among the groups, we analyzed the free amino acid contents in the serum of yellow catfish. Our study indicated that dietary mixed plant protein source replacement significantly influenced the free amino acid profiles in the serum. Generally speaking, we found that total NEAA, total EAA, and total amino acid contents declined with increasing mixed plant protein replacement levels. Decreased serum free amino acids mean that yellow catfish have limited utilization of mixed plant protein. Similar to our results, Yuan et al. [45] demonstrated that 5% replacement of fish meal by cottonseed meal protein hydrolysate in feed significantly reduces the total essential amino acid content in the plasma of blunt snout bream. Yuan et al. [45] showed that soybean meal reduced free amino acid contents in the turbot plasma.

The antioxidant defense system was also dependent on nutrition [46]. In our study, dietary mixed plant protein replacement significantly influenced hepatic antioxidant responses of yellow catfish. We found that fish fed the control and RM10 diet possessed higher CAT and T-SOD activities and total antioxidant capacity, the higher *sod1*, *sod2*, *cat*, and *gpx1* mRNA levels and the lowest MDA content. SOD and CAT are considered as important defense components against the free radicals in vertebrates [47], and MDA is usually as a biomarker to assess oxidative stress [48]. Similarly, other studies pointed out that high rapeseed meal levels reduced antioxidant capacity and increased oxidative stress in fish [9, 24, 38, 49, 50]. The reduced antioxidant enzyme activities after high plant protein inclusion may be due to the downregulation of mRNA expression of antioxidant genes. The reduced antioxidant enzyme activities will cause lipid peroxidation, which was confirmed by the significant MDA increase in yellow catfish fed increasing mixed plant protein levels. Again, the increased ANFs contents induced by dietary plant protein inclusion are potential stressors which adversely affect oxidative stress of fish. Studies suggested that the expression of antioxidant enzymes was controlled by Nrf2-Keap1 pathway [51]. Meantime, we found that the gene expression of *keap1* was declined, but the gene expression of *nrf2* was increased with the increment of dietary mixed plant protein levels; moreover, compared to other three groups, yellow catfish fed the control and RM10 diet had lowest Nrf2 protein expression but the highest Keap1 protein expression. Nrf2, an important transcriptional factor, regulates the expression of many antioxidative genes through the direct binding to the antioxidant response element (ARE) of target genes' promoters [12, 52]. Keap1 can bind with Nrf2 protein, prevents its translocation to the nucleus, and promotes its ubiquitination-proteasomal degradation [12, 53]. Studies indicated that phenolic compounds can induce oxidative stress by regulating Keap1 and Nrf2 [9]. Thus, the depressed antioxidant capacity of yellow catfish fed high mixed plant protein diets could be attributable to the presence of ANFs in these ingredients. Downregulation of mRNA expression of the antioxidant genes may result from the inhibition of Nrf2 signaling in fish.

In fish, the immune status is closely linked with inflammation initiation and controlled by the inflammatory cytokines [54]. In the present work, dietary mixed plant protein replacement significantly influenced mRNA expression of genes relevant with inflammatory responses, and high proportion (more than 20%) of mixed plant protein in feed significantly increased proinflammatory cytokines of *TNF-α*, *il1β*, *il6*, and *il8* mRNA expressions. The *TNF-α*, *il1β*, *IL6*, and *IL8* are important proinflammatory cytokines that are used as biomarkers for the activation of inflammatory responses [17]. These cytokines synergistically act to mediate the resistance to infections by controlling pathogen replication within the cells [16, 55]. Studies have shown that the upregulation of TNF-*α* and *IL8* could increase the inflammatory response [56, 57]. Our study indicated that the *il10* and *tgfβ* mRNA expression was variable and could not be linked to dietary treatments. At present, we do not know the exact reason since the TGF-*β* and *il10* could inhibit the excessive activation of the immune responses [58].

The TOR pathway plays the key regulatory roles in protein synthesis in response to nutrients [59, 60]. The mTOR stimulates the protein synthesis through 4EBPs and S6Ks in fish and in mammals [61, 62]. S6Ks is located in the downstream of TOR pathway and control the cell growth via regulating the translation of eukaryotic initiation factor 4B (*eIF4B*) and ribosomal protein S6 (rpS6) [63]. Previous studies reported that amino acids regulated the gene expression of TOR pathway [64]. However, few reports were published about the relationship between the protein feedstuff and TOR pathway. In the present study, the mRNA levels of mTOR, s6*, s6k1*, *eif4b*, and *eif4e* tended to decline but *4ebp1* mRNA levels tended to increase with increasing replacement levels. The 4E-BP1 is one small-molecular weight translational repressor [63]. Zhou et al. [37] found that fish meal replacement with canola meal decreased hepatic TOR mRNA levels and regulated the *s6k1* mRNA expression of blunt snout bream. Wacyk et al. [65] reported that dietary soybean meal decreased hepatic *tor* gene expression in rainbow trout. Yuan et al. [45] found that dietary 5% and 7% cottonseed meal protein hydrolysate (CPH) decreased S6K1 mRNA expression, further confirming that replacing fish meal with high CPH levels depressed the protein synthesis by inhibiting the TOR pathway. He et al. [66] showed that replacing fish meal with high cottonseed protein concentrate decreased the growth performance and inhibited mTOR pathway in largemouth bass. Thus, the *tor* mRNA level decreased significantly when fish meal was replaced by high levels of mixed plant proteins in diets, which in turn upregulated the gene expression of 4E-BP1, inhibited mRNA translation and cell proliferation, and accordingly inhibited the growth. We found that the ratios of p-mTOR/mTOR and p-S6/S6 reduced with increasing dietary replacement levels, further confirming the inhibition of TOR pathway since studies suggested that these proteins were regulated at the phosphorylation level [67].

## 5. Conclusion

In conclusion, replacing 10% fish meal with mixed plant protein could significantly improve growth performance without adverse effects on feed utilization and health status of yellow catfish. The second-regression analysis based on SGR against dietary mixed plant protein level indicated that their optimal replacement level was 8.7%. Higher replacement levels of fish meal by mixed plant protein sources reduced growth performance, damaged the liver histology and induced oxidative stress and inflammatory responses, and inhibited mTOR pathway. These findings provided a reference for the application of cottonseed meal and rapeseed meal in the diets for yellow catfish and other fish species.

## Figures and Tables

**Table 1:** Ingredients and proximate compositions of the experimental diets (g kg^−1^ dry matter).

Ingredients(g kg^−1^)	Control	RM10	RM20	RM30	RM40
Fish meal^a^	250	225	200	175	150
Chicken meat meal^a^	200	200	200	200	200
Soybean meal^a^	120	120	120	120	120
Peanut meal^a^	50	50	50	50	50
Wheat starch ^a^	161.75	161.75	161.75	161.75	161.75
Corn gluten meal^a^	80	80	80	80	80
Rapeseed meal^a^	0	24.9	51.7	78.6	105.5
Cottonseed meal^a^	0	14.6	27.6	40.6	53.6
Corn oil	20	18.68	17.34	15.99	14.65
Fish oil	10	12.04	14.09	16.13	18.18
Ascorbyl-2-polyphosphate^b^	10	10	10	10	10
Vitamin premix^c^	5	5	5	5	5
Mineral premix^d^	5	5	5	5	5
Choline^b^	5	5	5	5	5
Ca(H_2_PO_4_)_2_·H_2_O^b^	10	11.75	13.5	15.25	17
Y_2_O_3_	0.5	0.5	0.5	0.5	0.5
Cellulose	72.75	55.78	38.52	21.18	3.82
*Proximate analysis (% dry matter basis)*					
Dry matter	94.6	94.5	94.6	94.6	94.6
Crude protein	44.0	43.9	43.7	44.0	44.0
Crude lipid	9.43	8.85	8.82	8.96	9.00
Ash	8.79	8.83	8.77	8.88	8.63
Free gossypol (mg/kg)	ND	24.77	41.49	56.75	78.10

ND: not detectable. ^a^ Supplied by Zhanjiang Yuehai Feed Co., Ltd. (Zhanjiang, China); fish meal, 69.95% crude protein and 9.03% crude lipid; chicken meat meal, 63.42% crude protein and 12.49% crude lipid; soybean meal, 47.16% crude protein and 1.67% crude lipid; peanut meal, 50.79% crude protein and 7.21% crude lipid; wheat starch, 11.25% crude protein and 1.69% crude lipid; corn gluten meal, 60.69% crude protein and 3.81% crude lipid; rapeseed meal, 40.64% crude protein and 3.98% crude lipid; cottonseed meal, 51.89% crude protein and 3.29% crude lipid. ^b^Supplied by Shanghai Hanhong Chemical Co., Ltd. (Shanghai, China). ^c^Vitamin premix (mg or IU per kg diet): retinylacetate, 10000 IU; cholecalciferol, 1000 IU; all-rac-a-tocopheryl acetate, 30 IU; menadione nicotinamide bisulfite, 7; thiamine hydrochloride, 6; riboflavin, 3; pyridoxine hydrochloride, 12; D-calcium pantothenate, 30; niacin, 50; biotin, 1; folic acid, 6; cyanocobalamine, 0.03. ^d^Mineral mixture (mg per kg diet): Ca(H_2_PO_3_)_2_·H_2_O, 1000; FeSO_4_·7H_2_O, 40; ZnSO_4_·7H_2_O, 40; MnSO_4_.H_2_O, 40; CuSO_4_·5H_2_O, 2; CaIO_3_·6H_2_O, 3; Na_2_SeO_3_, 0.05; CoSO_4_, 0.05.

**Table 2:** Primers used for quantitative real-time PCR analysis.

Genes	Forward primer (5′-3′)	Reverse primer (5′-3′)	Accession no.
*mTOR*	GCCGATTTGCCAACTACCT	ACTCCAGAGCCCGCTTCAC	XM_027166728
s6	GCAAGCTCTTCAACCTGTCC	TTTCTCTTTGGCCTCCTTCA	XM_027144105
*s6kb1*	GTCCCGATGACTCCACACTT	ACTGGACTCACTGGCGTTCT	KY072932
*eif4b*	AACCACCCTCTCCAGGAAGT	CGTGGACAATCTCCCTTGTT	XM_027144909
*4ebp1*	GGGACTCTGTTTAGCACCAC	AAACTGGGCGTCTTCACC	XM_027140104
*eif4e*	TGACGAAGCCAGTGAAGATG	GTTTTTGACGGGGAGATTCA	XM_027142082
*sod1*	CCTCAAAGGCACAGGAGAAG	AATCGGCAGTCACATTACCC	XM_027171881
*sod2*	TCATGCAGCTTCACCATAGC	CTGTGGTTCTCCTCCACCAT	XM_027166181
*gpx1*	GATCAGACAGAGGCGTGACA	GCTGAGCTCTGCTGTACGTG	MN062284
*Cat*	CGTTTAGCCGCTACTGATCC	CTGGAATCAGGGGGTAGTCA	KX455919
*nrf2*	GTGAAGGGGGAAACACAGA	GCTCGTCCATGTCAGAGTCA	KX455917
*keap1*	CGAATCGCATTGCTTAGTCA	CGGAACTAAAAGGTGGTGGA	XM_027133478
*il1β*	CTGTGTGTTTGGGGATTGTG	GGGTATTTCACCGACTCGAA	MF770571
*il6*	ATGCCTCACCTAGAGCAGGA	GTGAAGCTGTGCAGAATGGA	XM_027176013
*il8*	GACTGCGATGCTTTGTGAAG	TCAGGCAGACCTTCATTCCT	KY218792
*tnfα*	TCTGCTTCACCATCTTCGTG	GGCACCAGCTTCTTGACTTC	XM_027165847
*tnfβ*	CGGTGGTCATCTCACACATC	CAGCTCAAACACCCCAAAAT	XM_027146388
*il10*	TCCTGCTTTCTTTGCTGACA	GAATAGTGCGGTGTCCAGGT	KY218793
*tgfβ*	ACAGGTCCAAAGGTTGATGG	CACATCCACAAGAAGCTGGA	XM_027154377
*β*-*Actin*	GGACTCTGGTGATGGTGTGA	CTGTAGCCTCTCTCGGTCAG	EU161066
*rpl7*	GGCAAATGTACAGGAGCGAG	GCCTTGTTGAGCTTGACGAA	KP938522
*Hprt*	ATGCTTCTGACCTGGAACGT	TTGCGGTTCAGTGCTTTGAT	KP938523
*Tuba*	TCAAAGCTGGAGTTCTCGGT	AATGGCCTCGTTATCCACCA	KP938526
*b2m*	GCTGATCTGCCATGTGAGTG	TGTCTGACACTGCAGCTGTA	KP938520
*Ubce*	TCAAGAAGAGCCAGTGGAGG	TAGGGGTAGTCGATGGGGAA	KP938524
*Tbp*	AGCAAAGAGTGAGGAGCAGT	ACTGCTGATGGGTGAGAACA	KP938525
*Gapdh*	TTTCAGCGAGAGAGACCCAG	ATGACTCTCTTGGCACCTCC	KP938521
*18* s *rRNA*	AGCTCGTAGTTGGATCTCGG	CGGGTATTCAGGCGAGTTTG	KP938527
*Elfa*	GTCTGGAGATGCTGCCATTG	AGCCTTCTTCTCAACGCTCT	KU886307

Abbreviations: *mtor*: mammalian/mechanistic target of rapamycin; *s6*: ribosomal protein S6; *s6kb1*: ribosomal protein S6 kinase beta 1; *eif4b*: eukaryotic translation initiation factor 4B; *eif4e*: eukaryotic translation initiation factor 4E; *4ebp1*: eukaryotic translation initiation factor 4E binding protein 1; *sod1*: superoxide dismutase 1; *sod2*: superoxide dismutase 2; *gpx1*: glutathione peroxidase 1; cat: catalase; nrf2: nuclear factor erythroid 2-related factor 2; keap1: kelch like ECH associated protein 1; *il1β*: interleukin 1 beta; *il6*: interleukin 6; *il8*: interleukin 8; *tnfα*: TNF alpha induced protein; *tnfβ*: tumor necrosis factor b; *il10*: interleukin 10; *tgfβ*: TGFB induced factor homeobox 1; *elfa*: translation elongation factor; *gapdh*: glyceraldehyde-3-phosphate dehydrogenase; *hprt*: hypoxanthine-guanine phosphoribosyl transferase; *rpl7*: ribosomal protein L7; *tbp*: TATA-box-binding protein; *tuba*: tubulin alpha chain; *ubce*: ubiquitin-conjugating enzyme.

**Table 3:** Effects of mixed plant protein substitute for fish meal on growth performance and feed utilization of yellow catfish.

Items	Control	RM10	RM20	RM30	RM40
IBW	2.40 ± 0.01	2.39 ± 0.02	2.42 ± 0.02	2.35 ± 0.01	2.33 ± 0.02
FBW	24.7 ± 0.5^b^	27.9 ± 1.3^c^	23.6 ± 0.5^b^	22.6 ± 1.1^b^	18.5 ± 0.6^a^
Survival^2^	95.6 ± 1.1	96.7 ± 0.0	96.7 ± 0.0	92.2 ± 1.1	95.6 ± 2.9
FI^3^	2.39 ± 0.06^a^	2.34 ± 0.09^a^	2.52 ± 0.02^a^	2.44 ± 0.11^a^	2.77 ± 0.1 ^b^
WG^4^	926.1 ± 13.3^b^	1064.7 ± 64.8^c^	873.6 ± 17.2^b^	859.7 ± 49.6^b^	695.0 ± 21.1 ^a^
SGR^5^	3.32 ± 0.02^b^	3.50 ± 0.08^c^	3.25 ± 0.03^b^	3.23 ± 0.07^b^	2.96 ± 0.04^a^
FCR^6^	1.03 ± 0.03^a^	0.98 ± 0.04^a^	1.09 ± 0.01^a^	1.07 ± 0.06^a^	1.27 ± 0.05^b^

^1^Values are means ± SEM (*n* = 3), and the values with different superscript letters in the same row are significantly different (*P* < 0.05); IBW (g/fish): initial mean body weight; FBW (g/fish): final mean body weight. ^2^Survival = 100 × final fish number/initial fish number; ^3^feed intake (FI, %day^−1^) = 100 × dry feed intake/[(*W*_*t*_ + *W*_0_)/2 × *t*]; ^4^Weight gain (WG, %) = 100 × (*W*_*t*_ − *W*_0_)/*W*_0_; ^5^Specific growth rate (SGR, %day^−1^) = (Ln *W*_*t*_ − Ln W_0_) × 100/t; ^6^ Feed conversion ratio (FCR) = (dry feed weight, g)/(wet weight gain, g);.

**Table 4:** Effects of dietary mixed plant protein substitute for fish meal on the proximate composition (percentage of live weight) and free gossypol content (mg/kg) in the liver of yellow catfish.

Item	Control	RM10	RM20	RM30	RM40
Moisture	74.2 ± 0.2	72.4 ± 0.6	74.3 ± 0.9	74.2 ± 0.2	72.1 ± 1.3
Crude protein	12.8 ± 0.4^bc^	13.2 ± 0.4^c^	11.4 ± 0.3 ^a^	11.7 ± 0.3^ab^	11.1 ± 0.4^a^
Crude lipid	6.5 ± 0.18^a^	6.61 ± 0.09^ab^	7.04 ± 0.06^c^	6.9 ± 0.07^bc^	7.21 ± 0.14 ^c^
Free gossypol (mg/kg)	0.00 ± 0.00^a^	11.5 ± 1.8^b^	19.6 ± 1.3 ^c^	26.9 ± 2.1^d^	34.4 ± 3.7^e^

Values are means ± SEM (*n* = 3), and the values with different superscript letters in the same row are significantly different (*P* < 0.05).

**Table 5:** Serum free amino acid concentration (nmol/ml) of juvenile yellow catfish fed the experimental diets.

	Control	RM10	RM20	RM30	RM40
EAA					
Met	30.36 ± 2.75^ab^	44.55 ± 0.42^c^	33.87 ± 2.29^b^	31.73 ± 4.51^ab^	23.25 ± 2.42^a^
Lys	342.3 ± 8.3^c^	294.1 ± 5.4^b^	310.1 ± 18.57^b^	300.9 ± 6.1^b^	195.2 ± 4.0^a^
Arg	364.2 ± 5.1^c^	354.3 ± 9.7^abc^	330.3 ± 6.1^a^	334.6 ± 11.6^ab^	357.0 ± 3.9^bc^
Val	151.3 ± 4.7^b^	149.6 ± 2.4^b^	138.2 ± 2.2^b^	139.9 ± 5.5^b^	108.1 ± 6.8^a^
His	218.9 ± 10.7^c^	180.6 ± 2.9^b^	183.3 ± 3.3^b^	179.1 ± 8.0^b^	143.4 ± 7.1^a^
Leu	192.4 ± 10.4^bc^	208.6 ± 8.2^c^	190.5 ± 6.9^abc^	181.6 ± 5.7^ab^	165.5 ± 7.4^a^
Ile	89.11 ± 3.38^b^	98 ± 6.58^b^	80.56 ± 6.04^b^	83.52 ± 6.71^b^	54.07 ± 7.34^a^
Phe	71.34 ± 5.16^b^	73.01 ± 0.87^b^	72.99 ± 1.98^b^	72.25 ± 4.68^b^	47.65 ± 4.19^a^
Thr	142.7 ± 9.3^b^	143.6 ± 0.3^b^	131.1 ± 4.2^b^	140.2 ± 6.1^b^	93.6 ± 5.5^a^
NEAA					
Ser	147.59 ± 6.62 ^b^	147.85 ± 6.51^b^	129.15 ± 3.25^b^	127.87 ± 4.83^b^	90.48 ± 10.86^a^
Ala	240.0 ± 11.0^c^	215.3 ± 3.4^bc^	198.6 ± 5.5^b^	210.2 ± 11.2^b^	140.0 ± 5.2^a^
Gly	238.5 ± 1.9^b^	228.9 ± 3.4^b^	234.2 ± 5.9^b^	238.1 ± 7.4^b^	128.4 ± 6.1^a^
Tyr	96.56 ± 3.7^b^	93.61 ± 4.93^b^	84.67 ± 6.37^b^	82.22 ± 5.94^b^	37.54 ± 3.92^a^
Asn	93.12 ± 3.96^c^	78.93 ± 2.36^b^	97.09 ± 3.32^c^	80.29 ± 3.25^b^	63.6 ± 5.7^a^
Glu	122.4 ± 2.2^cd^	134.4 ± 1.6^d^	94.2 ± 2.0^b^	110.2 ± 9.2^c^	58.8 ± 5.8^a^
Pro	45.31 ± 3.78^b^	45.25 ± 6.88^b^	49.69 ± 2.38^d^	40.85 ± 0.64^b^	12.45 ± 0.32^a^
Cys	26.06 ± 0.09	28.23 ± 3.54	35.64 ± 4.44	24.95 ± 3.43	25.94 ± 2.89
Asp	36.87 ± 4.2^c^	24.04 ± 1.16 ^ab^	27.78 ± 1.11 ^b^	20.48 ± 1.32 ^ab^	16.66 ± 2.21 ^a^
*Σ*NEAA	1046.4 ± 30.5 ^c^	996.5 ± 6.3 ^bc^	951.0 ± 28.9 ^bc^	935.2 ± 41.5 ^b^	573.8 ± 22.5 ^a^
*Σ*EAA	1602.6 ± 26.3^c^	1546.3 ± 9.6 ^bc^	1470.8 ± 23.4 ^b^	1463.76 ± 44.0^b^	1187.6 ± 26.1^a^
*Σ*AA	2649.0 ± 55.6^c^	2542.8 ± 7.3^bc^	2421.8 ± 87.1 ^b^	2398.9 ± 79.9^b^	1761.5 ± 46.1^a^

Values are means ± S.E.M of three replications. Means in the same line with different superscripts are significantly different (*P* < 0.05). *Σ*EAA: total essential amino acids. *Σ*NEAA: total nonessential amino acids. *Σ*AA: total amino acids.

## Data Availability

Data will be available with reasonable requirement with corresponding author.
